# Cowpea (*Vigna unguiculata* L. Walp) hosts several widespread bradyrhizobial root nodule symbionts across contrasting agro-ecological production areas in Kenya

**DOI:** 10.1016/j.agee.2017.12.014

**Published:** 2018-07-01

**Authors:** Samuel Mathu Ndungu, Monika M. Messmer, Dominik Ziegler, Hannes A. Gamper, Éva Mészáros, Moses Thuita, Bernard Vanlauwe, Emmanuel Frossard, Cécile Thonar

**Affiliations:** a Institute of Agricultural Sciences, ETH Zurich Plant Nutrition group Eschikon 33, CH-8315 Lindau, Switzerland; b International Institute of Tropical Agriculture (IITA), c/o ICIPE Campus, P.O. Box 30772-00100 Nairobi, Kenya; c Research Institute of Organic Agriculture (FiBL), Ackerstrasse 113, CH-5070 Frick, Switzerland; d Mabritec AG, Lörracherstrasse 50, CH-4125 Riehen, Switzerland; e Current address: AgroBioChem Department, Gembloux Agro-Bio Tech, University of Liège, B-5030 Gembloux, Belgium

**Keywords:** *Bradyrhizobium* distribution, Cowpea (*Vigna unguiculata* L. Walp), MALDI-TOF MS, Agro-ecology

## Abstract

Cowpea (*Vigna unguiculata* L. Walp.) is an important African food legume suitable for dry regions. It is the main legume in two contrasting agro-ecological regions of Kenya as an important component of crop rotations because of its relative tolerance to unpredictable drought events. This study was carried out in an effort to establish a collection of bacterial root nodule symbionts and determine their relationship to physicochemical soil parameters as well as any geographical distributional patterns. *Bradyrhizobium* spp. were found to be widespread in this study and several different types could be identified at each site. Unique but rare symbionts were recovered from the nodules of plants sampled in a drier in-land region, where there were also overall more different bradyrhizobia found. Plants raised in soil from uncultivated sites with a natural vegetation cover tended to also associate with more different bradyrizobia. The occurrence and abundance of different bradyrhizobia correlated with differences in soil texture and pH, but did neither with the agro-ecological origin, nor the origin from cultivated (n = 15) or uncultivated (n = 5) sites. The analytical method, protein profiling of isolated strains by Matrix-Assisted Laser Desorption Ionization Time-of-Flight Mass Spectrometry (MALDI-TOF MS), provided higher resolution than 16S rRNA gene sequencing and was applied in this study for the first time to isolates recovered directly from field-collected cowpea root nodules. The method thus seems suitable for screening isolate collections on the presence of different groups, which, provided an appropriate reference database, can also be assigned to known species.

## Highlights

• Bradyrhizobial root nodule symbionts of cowpea (*Vigna unguiculata* L. Walp) are diverse and widespread.
• Soil texture and pH seem to influence the occurrence and abundance of the different bradyrhizobial root nodule symbionts of cowpea.
• MALDI-TOF MS protein mass profiling of rhizobial isolates provides higher resolution than 16S rRNA gene sequencing.

## 1 Introduction

Cowpea (*Vigna unguiculata* L. Walp) is an important food legume and an essential component of sustainable cropping systems in the sub humid tropics and, generaly, dry regions across the globe (Singh et al., 2002). In Kenya, it is grown in the drier eastern area around Mbeere, as well as, in the humid coastal area around Kilifi, where it makes up an important part of the diet of small-scale farmers (Kimiti et al., 2009).

Cowpea is considered promiscuous in its association with root nodule-colonising bacteria, so-called rhizobia. It was shown to establish symbioses with several species and genera of the phyla Alpha- and Betaproteobacteria (de Souza Moreira et al., 2006; Pule-Meulenberg et al., 2010). Symbiotic association with effective rhizobia is a prerequisite to attain maximal benefits from symbiotic N_2_ fixation. Symbiotic N_2_ fixation can compensate for missing soil nitrogen (N) and thus potentially save costly mineral N fertilizer (Guimarães et al., 2012; Rashid et al., 2012). Rhizobial inocula for inoculating legumes increasingly account for differences in symbiotic specificity and effectivity, two parameters that are often correlated (Batista et al., 2015). Yet, the agro-ecological origin of rhizobial inoculants and thus most probably edaphic and climatic adaptation are often not considered sufficiently to make inoculation successful. A variety of biotic and abiotic factors, such as host plant, cultivation history, drought, soil pH, salinity, mineral nutrient availability, soil organic carbon content and texture, are known to affect rhizobial diversity and distribution (Giller, 2001; Law et al., 2007; Grönemeyer et al., 2014; Wade et al., 2014). However, collections of strains for inoculum development linked to such information are still rare.

In order to find adapted strains with a chance to establish and persist after inoculation, it is thus important to know their geographical and ecological distribution and physicochemical soil requirements. Differences in strain occurrence and abundance depending on these environmental parameters can provide this information, if based on observations across many sites.

To discriminate and identify rhizobial strains, there is a wide array of mostly DNA-based analytical tools. Strains can be discriminated based on Polymerase Chain Reaction-Restriction Fragment Length Polymorphisms (PCR-RFLP) and assigned to taxa by phylogenetic analysis of nucleotide sequences, such as those of the 16S rRNA and 23S rRNA genes, or the 16S-23S rRNA intergenic transcribed spacer (IGS) (Krasova-Wade et al., 2003; Qian et al., 2003; Pule-Meulenberg et al., 2010). Recently, however, a rapid high-throughput assignment technique emerged that relies on the cellular protein profiles, as characterized by Matrix Assisted Laser Desorption/ionization Time of Flight (MALDI-TOF) Mass Spectrometry (MS) (Ferreira et al., 2011; Ziegler et al., 2012). This protein profile-based approach to strain identification is yet mostly applied in the clinical diagnostics, where it has partially replaced biochemical assays and DNA-based discrimination and identification methods (Singhal et al., 2015). However, MALDI-TOF MS protein profiling has also already been used to assign *Bradyrhizobium* symbionts isolated from root nodules of cowpea, siratro (*Macroptilium atropurpureum* (DC.) Urb.) and soybean (*Glycine max* (L.) Merr) to species (Ziegler et al., 2012).

The objectives of the present study were to (i) determine the root nodule-colonising rhizobia of cowpea in the field (i.e. at cultivated sites) and of cowpea raised in pots with soil from uncultivated sites in two contrasting agro-ecological regions of Kenya and (ii) assess how their occurrence and abundance relates to geography, cultivation of cowpea and other grain legumes as well as physicochemical soil parameters at the collection sites. This was done with the intention to compile a collection of isolates with known edaphic requirements to develop site condition-adapted inoculants for cowpea.

## 2 Materials and methods

### 2.1 Study regions and sites, soil and root nodule sampling and soil analyses

Root nodule and soil samples were collected in the two most important cowpea-growing areas of Kenya, belonging to two different agro-ecological regions, the area around Mbeere (lower midland) and Kilifi (coastal lowland), about 600 km distance apart (Table 1). The Mbeere area is a dry and high elevation area and the Kilifi area is next to the coast with a more humid and buffered climate (i.e. less extreme temperatures and drought spells). The annual rainfall follows a bi-modal pattern, allowing for two cropping seasons in both agro-ecological regions (Table 1). The Mbeere area is considered as part of the Arid and Semi-Arid Lands (ASALS) of Eastern Kenya and is characterized by frequent droughts due to erratic and unreliable rainfall, while the Kilifi area is hot and humid throughout the year (Jaetzold et al., 2006). The soils around Mbeere are predominantly rhodic and orthic ferralsols, thus well drained, moderately deep to deep dark red to yellowish red, friable sandy-loams, while the soils around Kilifi are mostly cambisols, phaeozems, and rendzinas, less weathered clayey soils with high amounts of organic matter in the topsoil (Jaetzold et al., 2006). Information on soil texture for all study sites is listed in Table S1.

**Table 1:** Geographical, climatic and physicochemical soil characteristics of the two agro-ecological study regions in the eastern Mbeere and coastal Kilifi areas. Parameter ranges and if applicable averages of 15 cultivated and 5 uncultivated sites per region are listed. Composite soil samples were taken to a depth of 15 cm.

	Mbeere	Kilifi
Altitude (m a.s.l)	1049–1209	61–271
Agro-ecological zone	Lower midland	Costal lowland
Mean annual temp. (°C)	15–30	22–34
Mean rainfall (mm y^−1^)	640–1110	380–1230
Short rain	October–January	October–December
Long rain	March–June	April–July

Soil properties	Cultivated			Uncultivated			Cultivated			Uncultivated		
	Mean	Min	Max	Mean	Min	Max	Mean	Min	Max	Mean	Min	Max
pH (H_2_O)	6.4	5.4	7.2	6.4	6.1	6.6	5.9	5.1	6.6	6.2	6.1	6.3
Total N^a^ (g kg^−1^)	0.9	0.2	1.5	1.0	0.6	1.5	0.7	0.3	1.3	0.7	0.4	1.3
Organic C^b^ (g kg^−1^)	9.3	2.1	15.3	10.2	5.5	14.8	6.6	2.7	12.8	7.5	3.9	12.8
P^c^res (mg kg^−1^)	4.03	0.88	28.19	1.38	1.09	2.18	2.49	0.92	17.50	4.36	0.57	18.04
Clay^d^ (g kg^−1^)	171	96	256	197	129	329	337	96	696	372	96	675
Sand^d^ (g kg^−1^)	680	504	804	628	404	764	471	144	904	520	145	884
Silt^d^ (g kg^−1^)	149	60	300	175	107	267	192	0	440	108	20	180

a Total N: Kjeldahl method (Bremner, 1960).

b Organic C: (Walkley and Black, 1934).

c Presin: P_i_ extracted with anion exchange resin membranes.

d clay, sand and silt: hydrometer method (Bouyoucos, 1962).

This field survey relied on a sampling scheme with 20 sites per agro-ecological region; 15 farmers’ fields (i.e. ‘cultivated sites’) with a history of cowpea cultivation and five sites with no prior history of crop production (i.e. ‘uncultivated sites’). The geographical co-ordinates of each sampled site are also listed in Table S1. The sites differed in topography, microclimate and soil physico-chemical properties (Table 1). The sites were selected in consultation with regional agricultural extension officers, knowing smallholder farmers who grew cowpea and were willing to allow root nodule sampling. The sampling areas around Kilifi and Mbeere covered about 33 km^2^ and 17 km^2^, respectively. None of the selected sites had a previous known history of inoculation with rhizobia (pers. com. with farmers by Samuel Mathu Ndungu). Nodule and soil samples of cultivated fields were collected at the flowering stage that was for Mbeere in May 2013, and for Kilifi in August 2013 due to later planting time. At uncultivated sites only soil samples could be collected, which were used for physico-chemical characterisation and trapping indigenous rhizobia with cowpea in pot cultures (Zilli et al., 2004; Silva et al., 2012).

The soils were sampled to a depth of 15 cm by pooling five cores into a composite sample per site. After air-drying, the soil samples were passed through a 2 mm sieve before the chemical properties were determined by the MEA Ltd. soil and tissue testing laboratories (Nakuru, Kenya) and the texture was determined by the International Centre for Tropical Agriculture (CIAT) soil laboratory (Nairobi, Kenya). The measured parameters were total nitrogen, based on the Kjeldahl procedure (Bremner, 1960), organic carbon, using the method of Walkley and Black (1934), pH (H_2_O), and soil texture, using the hydrometer method (Bouyoucos, 1962). The bio-available inorganic phosphorus (P) was measured in Zurich (Plant Nutrition Group, ETH) as resin-extractable P (Pres) and was determined in triplicate by extraction with anion exchange resin membranes. In brief, 2–3 g moist soil was shaken with 30 ml of double-distilled water and two resin strips of 3 cm × 2 cm (BDH Laboratory Supplies product 55164 2S, Poole, England) for 16 h at 160 rpm on a horizontal shaker. The membranes were rinsed with water, and P was eluted with 0.1 M NaCl/HCl, followed by colorimetric concentration measurements, using malachite green (Ohno and Zibilske, 1991).

### 2.2 Cowpea cropping system of the sampled fields

Cowpea is the main crop during the short and long rainy seasons in the Kilifi and Mbeere areas, which leads to a nearly continuous presence of cowpea as a host of rhizobia. In both regions farmers grow additionally common bean (*Phaseolus vulgaris* L.), green gram (*Vigna radiata* (L.) Wilczek.), and pigeonpea (*Cajanus cajan* (L.) Millsp.) (Table S1). These also form root nodules with *Rhizobium* spp. and *Bradyrhizobium* spp. as symbionts and may thereby increase the diversity of cowpea-nodulating rhizobia. Cowpea can be grown as a sole crop, but is mostly intercropped with maize (*Zea mays* L.), sorghum (*Sorghum bicolor* (L.) Conrad Moench) and pearl millet (*Pennisetum glaucum* (L.) R.Br) and in the coastal region of Kenya also with cassava (*Manihot esculenta* Crantz) (Table S1).

In both agro-ecological regions soils are infertile because of nutrient depletion as a consequence of little mineral and organic fertilizer use by the resource-poor farmers. Typical cropping involves alternating rows of cereals, such as maize, sorghum and millet, and legumes, such as common bean, green gram, pigeonpea, and cowpea, with the latter being the most dominant in both regions (Table S1).

### 2.3 Nodule collection in farmers’ fields and from trap culture plants

Root nodules were collected from cowpea plants in farmers’ fields, giving samples for the ‘cultivated sites’. At each site, five healthy cowpea plants were selected for uprooting and collection of nodules. Nodules were stored in McCartney glass vials with dehydrated silica gel for transport to the laboratory and storage at 4 °C until bacterial isolation.

To trap rhizobia from the soil samples of the ‘uncultivated sites’, two approaches were used: (1) trapping of rhizobia in 300 g plastic pots filled with a 2:1 (v:v) mixture of native soil and autoclaved quartz sand (grain size: 0.7-1.2 mm) planted with one cowpea plant (Zilli et al., 2004), or (2) sand cultures of 600 g pure autoclaved sand with seedlings inoculated with 5 g of air-dried native field soil. To isolate rhizobia from commercial Biofix inoculum (MEA Ltd. Nakuru, Kenya) as a reference (CBA), because this inoculum is recommended for cowpea in Kenya, one gram of inoculum was added close to a seedling growing in sand culture. For further reference, there was also a trap culture set up with soil from Burkina Faso from which strain BK1, an efficient strain, was isolated for future functional tests on symbiotic effectiveness (paper in preparation). Two cowpea cultivars commonly cultivated in the Mbeere and Kilifi regions, K80 (cultivar bred by Kenya Agricultural and Livestock Research Organization) and Black eyed pea (cultivar widely propagated by farmers themselves), were used as trap plants. Three pots were set up for each soil and cowpea cultivar. Seeds were surface sterilized by immersion in ethanol (70%; 30 s), hydrogen peroxide (2%; 2 min) and thorough rinsing with autoclaved water. Sterilized seeds were imbibed in water for 1 h, and subsequently germinated on moistened cotton wool in Petri dishes in a growth chamber at 28 °C in the dark for 24 h or until radicle emergence. Upon germination, seeds were transferred to the growth substrate to which Broughton and Dilworth’s N-free plant nutrient solution (Broughton and Dilworth, 1970) was added three times a week in alteration with water for the entire growth period of the plants. Conditions in the growth cabinet were set to 12 h of light from Grolux (1000 lm) and Sylvania white cool (5000 lm) lamps, and to 27/20 °C (day/night) temperature. The air humidity fluctuated between 60 and 70%. The plants were harvested for root nodule sampling 40 days after sowing, while still in the vegetative stage. Root systems were rinsed and the nodules detached for immediate surface sterilization (see next section) and storage in diluted glycerol (4:6, v:v glycerol: water) in 2 ml cryo-vials at −20 °C.

### 2.4 Strain isolation from root nodules

The dried root nodules from the field were rehydrated in sterile distilled water prior to surface-sterilization, while the nodules of the trap cultures were immediately surface-sterilized. After immersion in 70% ethanol for 30 s, nodules were immediately transferred to 3.85% NaOCl solution for 2 min before three thorough rinses in sterile distilled water. Each nodule was crushed in 50 μl of sterile 40% glycerol in a sterile 1.5 ml Eppendorf tube, using a sterile plastic pestle. A wire loop full of the nodule homogenate was dilution-streaked on Yeast extract Mannitol Agar (YMA) plates (Somasegaran et al., 1994). Plates were incubated in the dark at 28 °C for 3–5 days to allow for growth of *Bradyrhizobium* isolates. Single strain isolates were obtained by repeated further dilution-streaking of single colonies. Glycerol stocks for long-term storage at −80 °C were prepared in Yeast extract Mannitol (YM) broth supplemented with 20% (v:v) glycerol.

### 2.5 Discrimination of *Bradyrhizobium* strains

#### 2.5.1 Matrix-assisted laser desorption/ionization-time of flight (MALDI-TOF) mass spectrometry (MS) of bacterial cell lysates

In preparation for MALDI-TOF MS analysis, all isolated strains (Table S1) were sub-cultured on Modified Arabinose Gluconate (MAG) plates (Sadowsky et al., 1987; Van Berkum, 1990) for four days to get colonies with less exopolysaccharides, facilitating the spotting of the cells on the plates for cell lysis and analysis (pers. comm. Dominik Ziegler). All these sample preparation steps were done as described in Ziegler et al., 2012, Ziegler et al., 2015 by Mabritec AG, Switzerland (http://www.mabritec.com), a laboratory specialised on diagnostic analyses, using MALDI-TOF MS. In brief, bacterial samples were spotted in duplicate on MALDI steel target plates. Spots were overlaid with 1 μl of 25% formic acid, air-dried, and overlaid with 1 μl of alpha-cyano-4 hydroxycinnamic acid (CHCA; Sigma Aldrich, Buchs, Switzerland) in 33% acetonitrile (Sigma Aldrich), 33% ethanol and supplemented with 3% trifluoroacetic acid (TFA). After co-crystallisation at room temperature, target plates were introduced into the MALDI-TOF Mass Spectrometer Axima™ Confidence machine (Shimadzu- Biotech Corp., Kyoto, Japan) for sample analysis.

### 2.6 DNA extraction, PCR amplification and 16S rRNA gene amplicon sequencing of selected strains

Twenty-five representative strains of the protein profile-based similarity clusters (see Section 2.7.2) from cultivated and uncultivated sites of both agro-ecological regions were selected for additional 16S rRNA gene sequencing and phylotaxonomic identification. Genomic DNA was extracted from 2.2 ml of four day-old cultures in liquid YM broth, using the Nucleospin^®^ Microbial DNA Isolation Kit (Macherey Nagel GmbH & Co. KG, Germany). Cell lysis was mechanically enhanced by two 3 min runs in a TissueLyzer II (Qiagen, Valencia, CA, USA) swing mill at 30 Hz. DNA was recovered in 100 μl elution buffer and stored at −20 °C until PCR amplification.

For PCR amplification of nearly the entire 16S rRNA gene, forward primer 27F and reverse primer 1492R (Lane, 1991) were used. Reactions were carried out in 50 μl with 1 × Taq buffer, 0.6 Uμl^−1^ GoTaq^®^ DNA Polymerase (Promega, Madison, WI, USA), 0.2 mM dNTP, 3 mM MgCl_2_, 0.5 μM of each primer, 2 μl of genomic DNA and molecular grade water. The following amplification program was used: Initial denaturation at 95 °C (5 min), followed by 35 cycles of 95 °C (30 s), 56 °C (30 s), and 72 °C (1 min), and a final extension at 72 °C for 10 min. PCR amplicons were run on a 1.5% (w/v) agarose Sigma^®^ (Sigma-Aldrich Chemie Gmbh, Steinheim, Germany) gel, and visualised with the intercalating dye Midori^®^ Green (Nippon Genetics Europe GmbH, Germany) on a UV transilluminator. PCR amplicons were ethanol-precipitated and sent for Sanger sequencing with the primers 27F (Lane, 1991) and U1406R (Baker et al., 2003) to the company Microsynth (Balgach, Switzerland).

### 2.7 Strain grouping and taxonomic assignment

#### 2.7.1 MALDI-TOF mass spectral profiling

Binary matrices of the protein masses in the size range of 3000–12,000 Da were generated for each isolated rhizobial strain after aligning the profiles. 717 different protein masses were taken into account, which showed abundances higher than the background noise, using the Superspectra tool in the Spectral ARchive And Microbial Identification System (SARAMIS^™^) (Ziegler et al., 2015). Dice similarities (Dice, 1945) were used to prepare a pairwise similarity matrix for all the root nodule isolates. Using these similarities, the strains were clustered by multivariate neighbour joining in the Palaeontological Statistics Software Package, PAST v3.16 (Hammer et al., 2001), using a similarity cut-off of 60%. The resulting dendrogram was edited in the Tree Figure Drawing Tool, FigTree v1.4.2 (http://tree.bio.ed.ac.uk/software/figtree/).

#### 2.7.2 16S rRNA gene sequencing

The newly generated 16S rRNA gene sequences of the 25 representative strains of the different protein profile-based similarity clusters were aligned with CLUSTAL W in the software package MEGA 6.0 (Tamura et al., 2013). Also included were 17 reference sequences from the public sequence database NCBI GenBank of closely related type strains of *Bradyrhizobium* species. The multiple sequence alignment was trimmed to the 1058 aligned sites before Maximum Likelihood (ML) tree inference by PhyML in MEGA. Statistical support for tree branches and hence phylotaxonomic assignment of the new isolates to described species was obtained by bootstrapping the multiple alignment 1000 times. The new sequences were deposited in the European Nucleotide Archive of the European Molecular Biology Laboratory under the accession numbers LT618843-LT618867.

### 2.8 Statistical analyses

To check the sufficiency of strain sampling in the two agro-ecological regions and at cultivated and uncultivated sites, the numbers of recovered isolates belonging to five major protein spectral similarity clusters were subsampled in the freeware software Analytic Rarefaction (Holland, 2003). To reveal potential links to environmental parameters and regional and site class (‘cultivated’ and ‘uncultivated’) origin, the occurrence and abundance of the bradyrhizobial groups were correlated to the physico-chemical soil properties and the information about the origin of the isolates by ordination. A redundancy analysis (RDA) was run in the multivariate analysis software CANOCO v4.5 (Microcomputer Power, Ithaca, NY) (Lepš and Šmilauer, 2003), because an initial Detrended Correspondence Analysis (DCA) yielded a gradient length of 2.48 standard deviation units of the first ordination axis (Lepš and Šmilauer, 2003). The considered environmental parameters were the edaphic properties [organic carbon, total nitrogen (N), resin-extractable soil phosphorus (Pres), pH (H_2_O), clay and sand concentrations] and the origins from the two agro-ecological regions and cultivated and uncultivated sites, which were coded as dummy variables. After running the RDA with all environmental parameters and 499 unrestricted Monte Carlo permutations for significance testing, the non-significant factors were excluded from the species-environment biplot. The nodule samples of three out of the 40 study sites did not yield rhizobial isolates and thus had to be excluded from the analysis (one cultivated and one uncultivated site in Mbeere and one cultivated site in Kilifi). A further site from the Mbeere area had to be excluded from the RDA, because none of the isolated strains fell in one of the five groups of bradyrhizobia considered.

## 3 Results

### 3.1 Strain discrimination, grouping and taxonomic assignment

**Fig. 1:**
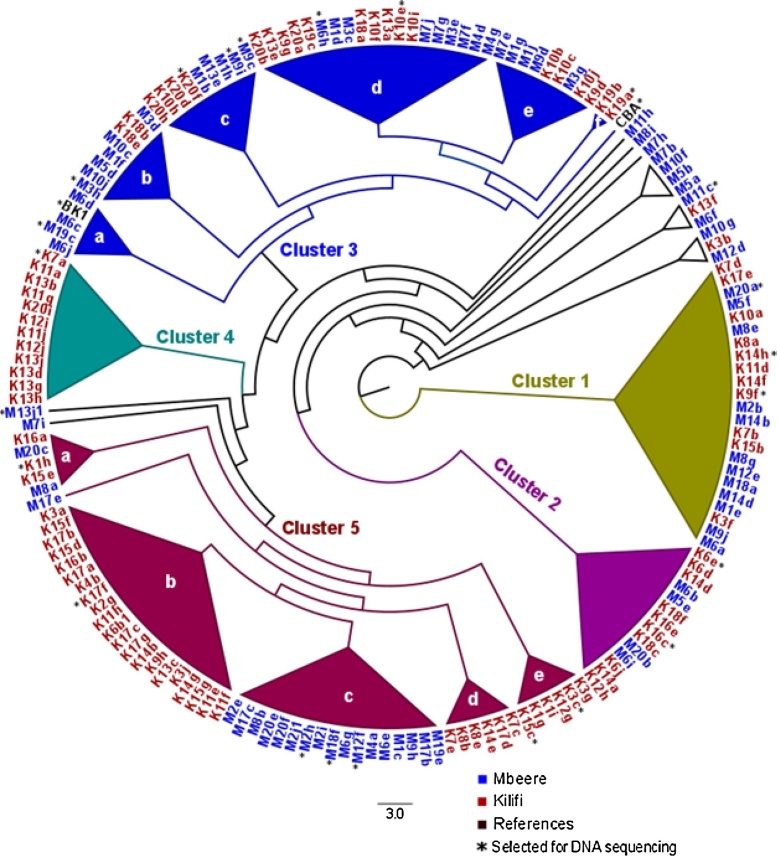
Dendrogram of an unsupervised hierarchical cluster analysis of rhizobial strains isolated from cowpea nodules based on Dice distances of mass spectral protein profiles of Matrix Assisted Laser Desorption/Ionization Time of Flight (MALDI-TOF) Mass Spectrometry (MS). Presence/absence of protein masses in the size range of mass-to charge ratio (*m*/*z*) 3000–12,000 was used. Strain origin is indicated in the strain identifiers (K: Kilifi, M: Mbeere). The strains 1–15 are from cultivated sites and the strains 16–20 from uncultivated sites. Reference strains were the isolates CBA from commercial Biofix inoculum (MEA Ltd. Nakuru, Kenya) and BK1 from a trap culture with soils from Burkina Faso. The scale bar shows the Dice distance in per cent.

**Fig. 2:**
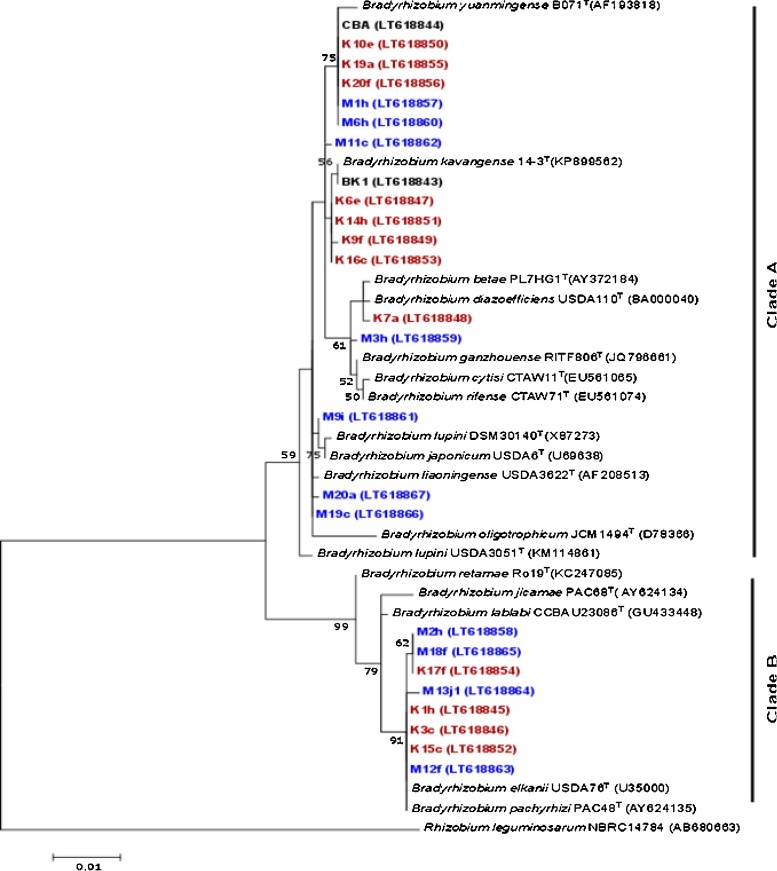
Maximum-likelihood phylogenetic tree based on near full-length 16S rRNA gene sequences of 25 *Bradyrhizobium* strains isolated from root nodules of cowpea and 17 species type strain sequences from the public databases. The tree is rooted with a further public database sequence of a type strain of a species of the genus *Rhizobium*. The statistical branch support values were obtained by bootstrap analysis (>50%) of 1000 resampled datasets. The strain identifiers indicate the geographical origin (K: Kilifi, M: Mbeere agroecological region). The strains 1–15 are from cultivated sites and the strains 16–20 from uncultivated sites. The sequence accession numbers are given in parentheses and the scale bar indicates the percentage of nucleotide substitutions. The colour coding follows that used in the dendrogram of Fig. 1.

One hundred and seventy one newly isolated *Bradyrhizobium* strains from the Kilifi and Mbeere agro-ecological regions including two reference strains CBA and BK1 could be characterized and identified based on the mass spectral profiles of their proteins. Their taxonomic assignment to the genus *Bradyrhizobium* was verified by near full-length 16S rRNA gene sequences, which enabled a grouping into two major phylogenetic clades A and B (Fig. 1, Fig. 2, Table S2). The analysis based on the similarity of the protein mass spectra recovered five distinctive clusters (1–5, Fig. 1); 15 *Bradyrhizobium* strains did not fall into these five clusters and remained separate. The cluster 3 consisted of six (a-f) and the cluster 5 of five sub-clusters (a-e, Fig. 1).

The 16S rRNA gene-based maximum likelihood tree (Fig. 2) showed that 17 of the 25 sequenced strains fell in clade A and eight in clade B. The grouping of strains, based on protein profile similarity and nucleotide sequence evolution showed most congruence for members of the *B. elkanii* group, represented by cluster 5 and clade B, respectively (Fig. 1, Fig. 2). Clade A comprises strains of the clusters 1, 2, 3, 4 and some further unclustered strains. The assignment of the clusters 1, 2, and 4 to the species groups *B. cytisi* and *B. japonicum* was inconsistent (Fig. 1, Fig. 2). Cluster 1 had 2 members identified as *B. kavangense* and one that grouped next to *B. liaoningense*; cluster 3 had 6 strains out of 10 including the reference strain CBA from *B. yuanmingense* and 1 that grouped next to *B. liaoningense*, 1 that grouped next to a subclade with *B. cytisi*, *B*. *rifense* and *B*. *ganzhouense*, as well as 1 that grouped next to a subclade with *B. lupini* and *B. japonicum*, *B*. *oligotrophicum* and 1 (BK1) from Burkina Faso that grouped next to *B*. *kavangense*. The sequenced representative strain of cluster 4 grouped next to a subclade with *B*. *diazoefficiens* and *B*. *betae* (Fig. 1, Fig. 2).

The remaining strains, besides the 171 *Bradyrhizobium* strains, belonged to other bacterial genera. Twenty four strains were affiliated to several *Rhizobium* spp., six to *Rhizobium radiobacter*, three to *Enterobacter cloacae*, one to *Staphylococcus warneri*, and nine strains remained unassigned (Table S2) in comparison to the reference library SARAMIS^™^ of Mabritec AG (Ziegler et al., 2015). Some strains such as *Staphylococcus warneri* could be surface contaminants as no nodulation tests were done.

### 3.2 Geographical and environmental distribution of different bradyrhizobia

**Fig. 3:**
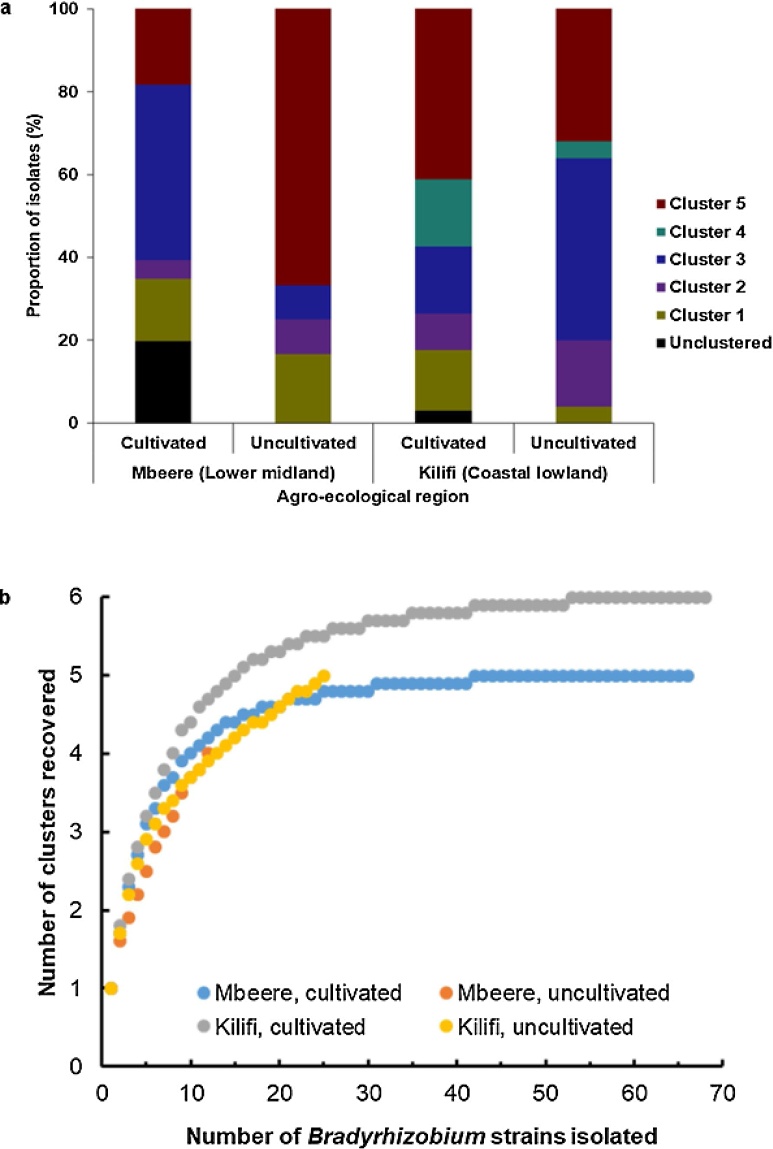
(a) Frequency of occurrence of five *Bradyrhizobium* isolate groups from root nodules of cowpea at cultivated and uncultivated sites in the agro-ecological regions around Mbeere and Kilifi in Kenya. The isolate grouping is based on similarity clustering of mass spectral protein profiles as determined by Matrix Assisted Laser Desorption/ Ionization Time of Flight (MALDI-TOF) Mass Spectrometry (MS) (Fig. 1). Sampling took place at 15 cultivated and 5 uncultivated sites in each agro-ecological region. (b) Rarefaction curves of the number of different groups of *Bradyrhizobium* in the two agro-ecological regions and cultivated and uncultivated sites (Fig. 1). Number of isolates considered: Mbeere, cultivated: 66, uncultivated: 12; Kilifi, cultivated: 68, uncultivated: 25.

As already evident from Fig. 1, Fig. 2, the different isolated bradyrhizobia did not show distinctive distributional patterns between the two agro-ecological regions and cultivated and uncultivated sites, except cluster 4, which only included strains isolated from the Kilifi area (Fig. 1, Table S.2) and two sub-clusters of cluster 5 also from the Kilifi area. Most clusters contained strains from cultivated and uncultivated sites of the two agro-ecological regions (Fig. 1). The frequency of occurrence also did not reveal a clear pattern when analysed for cultivated and uncultivated sites of the two agro-ecological regions (Fig. 3a). Exceptions were sub-cluster 3e, which consisted solely of strains from cultivated sites, albeit from both agro-ecological regions and sub-cluster 5e, which consisted solely of strains from cultivated sites of the Kilifi area (Fig. 1). Replicate strains from the same field often fell within different clusters, pointing at considerable strain richness at the field level and little evidence for over prevalence of certain clusters at particular sites. For example, in the Kilifi area, strains from site 13 were distributed in the clusters 2, 3, 4 and 5 and strains of site 1 of the Mbeere area were distributed across the clusters 1, 3 and 5 (Fig. 1).

In cultivated sites of Mbeere, strains of cluster 3 were most frequent (42%), followed by strains that did not cluster (20%) and strains of the clusters 5, 1 and 2 with frequencies of occurrence of 18%, 15% and 5%, respectively. Members of the clusters 5, 1, 2 and 3 were only recovered from nodules of trap plants grown in soil from uncultivated sites with frequencies of occurrence of 67%, 17%, 8% and 8% of the isolated strains, respectively (Fig. 3a). In cultivated sites from the Kilifi area, cluster 5 had the most strain representatives (41%). The clusters 4, 3, 1, 2 and unclustered types had the following frequency distribution of strain representatives: 16%, 16%, 15%, and 9%, respectively. In the uncultivated sites of Kilifi, most strains (44%) fell in cluster 3, followed by the clusters 5, 2, 4 and 1 with 32%, 16%, 4% and 4% of representative strains, respectively (Fig. 3a).

The effort in strain isolation seems to have been adequate for the cultivated sites for which taxon accumulation flattened off in the rarefaction analysis (Fig. 3b). The sampling of the uncultivated sites was, however, obviously not sufficient, although members of all, or most of the five similarity clusters were found at each uncultivated site. Taxon accumulation did not flatten, suggesting occurrence of more different bradyrhizobia at uncultivated than cultivated sites (Fig. 3b). According to the steepness of the taxon accumulation curves, the drier area around Mbeere, appears to host more different *Bradyrhizobium* strains than the coastal Kilifi area (Fig. 3b).

### 3.3 Relationship between rhizobial occurrence, abundance and edaphic properties

**Fig. 4:**
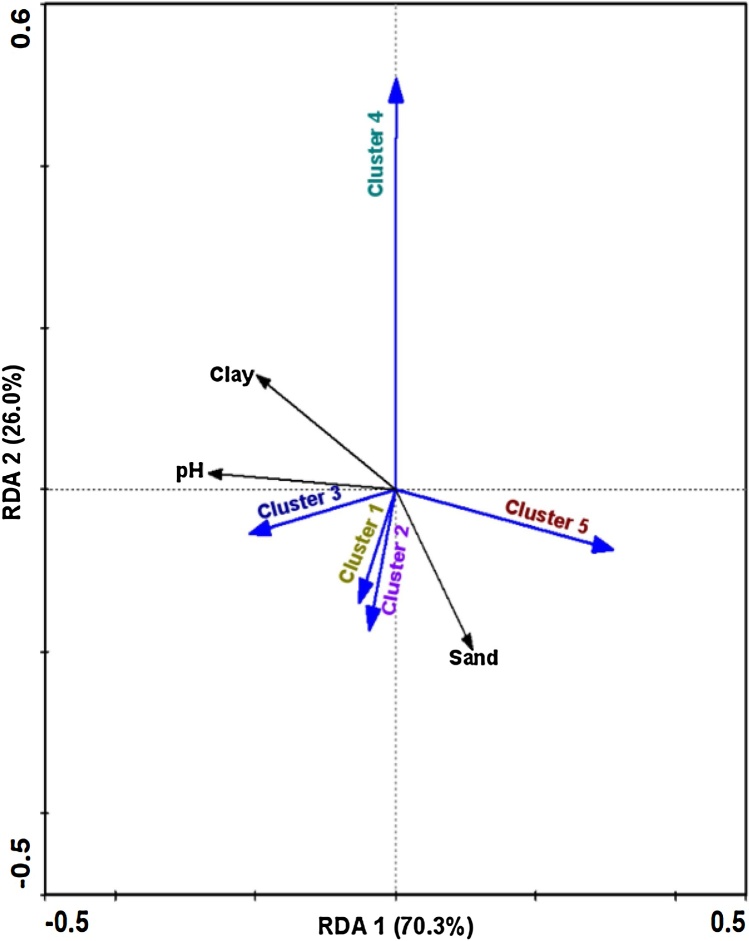
Correlative relationship between the occurrence of five similarity clusters of *Bradyrhizobium* root nodule isolates of cowpea and edaphic parameters as inferred by redundancy analysis (RDA). The biplot explains 96.3% of total variance in the dataset and is based on the presence/absence data of five similarity clusters of 156 *Bradyrhizobium* isolates, recovered from root nodules of cowpea plants growing in soil of 36 field sites. The vector sizes denote the strength of correlation and small angles indicate high correlation between environmental factors and/or cluster occurrence. Black arrows denote soil parameters, and blue arrows the *Bradyrhizobium* clusters, as characterized by similarity of MALDI-TOF MS protein mass spectra (Fig. 1, Fig. 3a). Percentages on the axes show the fraction of total variance explained. Only environmental parameters significantly correlated to the occurrence of the *Bradyrhizobium* clusters are shown. The pH (*P* < 0.05***), sand (*P* < 0.01**), and clay (*P* < 0.001*) concentrations, were those factors, which influenced *Bradyrhizobium* occurrence and symbiotic abundance. (For interpretation of the references to colour in this figure legend, the reader is referred to the web version of this article.)

Redundancy analysis of *Bradyrhizobium* abundance, geographical and site (‘cultivated’ and ‘uncultivated’) origin and the edaphic properties separated the clusters 1, 2 and 4 along a gradient of decreasing soil: sand content (Monte Carlo permutation test, *P* < 0.01**) and the clusters 3 and 5 along a gradient of high to low pH (*P* < 0.05***, Fig. 4). The clay content of the soil also affecting the communities of symbiotic bradyrhizobia (*P* < 0.001*) and must be related to soil pH and sand content (Fig. 4). The three parameters, sand content, pH and clay content explained 96.3% of the total variance in the community dataset (Fig. 4). Region, site, resin-extractable soil P, and the soil N and C contents did not significantly contribute to the rhizobial community composition and structure.

## 4 Discussion

### 4.1 Bacterial root nodule symbionts of cowpea of cultivated and uncultivated soil in Kenya

Using an isolation-based approach, this study confirmed representatives of the genus *Bradyrhizobium* to be the main root nodule symbionts of cowpea. There were neither major geographical patterns, nor a distinct partitioning between cultivated and uncultivated sites in the occurrence and abundance of five distinctive bradyrhizobial groups. Cowpea root nodules collected from the drier agro-ecological region around Mbeere and root nodules of plants inoculated with soil of uncultivated sites appeared to host more different bradyrhizobial symbionts than the more humid region around Kilifi and cultivated sites (Fig. 3b). There were only few site-, or region-specific bradyrhizobia and representatives of most or all five defined similarity clusters of bradyrhizobia were recovered from nodules of each site, pointing at little spatial community structuring in bradyrhizobia among agriculturally used areas in Kenya.

More different symbiotic *Bradyrhizobium* strains were found in the eastern, in-land, and drier agro-ecological region around Mbeere, where also some unique strains, such as those forming the clades I, II, III, IV, V, VI, VII and VIII (Table S2) occurred. It could thus be that these groups of bradyrhizobia are adapted to drier climatic conditions where protozoan predation in soil (Ramirez and Alexander, 1980) may be lower due to reduced connectivity via water films, or that the impact of agriculture and water drainage has not yet homogenised the bradyrhizobial community as much in this higher elevation and in-land area as in the costal agro-ecological region around Kilifi. Finding biogeographical distribution patterns may thus depend on the level of agricultural perturbation of local bacterial diversity, but may also depend on the resolution of the analytical method (Zhang et al., 2011; Koppell and Parker, 2012; Stępkowski et al., 2012). Higher richness of root nodule symbionts has already been reported for cowpea from low-rainfall areas in South Africa and Botswana by Law et al. (2007), which supports our notion that drier soils may be richer in rhizobia. Grönemeyer et al. (2014) also observed that the symbiotic communities of rhizobia from semi-arid sampling sites were more diverse than such from humid sites in Namibia. Similarly, Wade et al. (2014) reported a higher richness of cowpea-nodulating *Bradyrhizobium* strains from the drier north than the more humid south of Senegal. Alternatively, plant selectivity in association with rhizobia may be lower under drought than humid conditions.

### 4.2 Bradyrhizobia of cowpea and other tropical legumes

The present study confirmed members of the genus *Bradyrhizobium* to be the main symbionts of cowpea (Krasova-Wade et al., 2003; Krasova-Wade et al., 2006; Appunu et al., 2009; Pule-Meulenberg et al., 2010; Wade et al., 2014; Grönemeyer et al., 2015b), a bacterial genus well known to have its main distribution area in slightly to highly acidic soils of the tropics, and to be tolerant against fluctuations in soil temperature (Sprent et al., 2010). Similar to the finding in the present study, other studies have also reported cowpea to host some minority symbionts of the genus *Rhizobium* (Zhang et al., 2007; Steenkamp et al., 2008; Silva et al., 2012; Grönemeyer et al., 2014). Finding also other rhizobia, besides representatives of the genus *Bradyrhizobium*, supports the notion that cowpea, like groundnut (*Arachis hypogaea* L.), is promiscuous in its association with bacterial root nodule symbionts (Ibáñez et al., 2009; Silva et al., 2012). *Enterobacter* spp. have also previously been reported from nodules of cowpea (Leite et al., 2017), although surface contamination, or non-symbiotic persistence in the apoplast, cannot be excluded.

The symbiont richness from several different subgroups of the genus *Bradyrhizobium* of cowpea may relate to the fact that the plant genus *Vigna* to which cowpea belongs, originates from central Africa (Harlan, 1971; Lush and Evans, 1981), where also the diversification of its root nodule symbionts can be expected to have been the highest (Pule-Meulenberg, 2014). The rhizobial isolates of the root nodules of the trap cultures revealed that uncultivated sites may act as reservoirs of *Bradyrhizobium* diversity, which could be maintained by plant species diversity in the vegetation cover. However, overall, the dominant bradyrhizobia showed widespread occurrences at least at the level of resolution that MALDI-TOF MS protein profiling provides.

The recorded high richness of *Bradyrhizobium* strains within the cultivated sites may be attributable to several other legume crops, which farmers usually also cultivate; such as green gram and pigeonpea. These also associate with rhizobial symbionts that are shared with cowpea. Several studies have demonstrated that the diversity of rhizobia can be maintained when several different legumes are regularly part of cropping systems (Palmer and Young, 2000; López-López et al., 2013), as practiced by the smallholder farmers whose fields had been sampled for this study.

### 4.3 Relationship of bradyrhzobial occurrence and abundance with physicochemical soil properties

This study did not reveal strong patterns of biogeographical structuring of the symbiotic rhizobial communities of cowpea as lacking overall effects by the agro-ecological regions and site cultivation in the ordination analysis showed. It seems rather that several of the recorded groups of bradyrhizobia belong to globally distributed species groups, such as *B. elkanii*, *B. japonicum, B. diazoefficiens* and *Bradyrhizobium* sp. I. Many of the different bradyrhzobial groups were isolated from both agro-ecological study regions and cultivated as well as uncultivated sites. These bradyrhizobia must hence be ecological generalists, with high dispersal rates, probably promoted by agricultural soil management, such as ploughing. Lack of biogeographical community structuring and lack of major differences between cultivated and uncultivated sites may thus be explained by the fact that this study has been carried out in highly agriculturally used areas, where biotic homogenisation is happening, and from which rhizobia may spill over to uncultivated sites (Bell and Tylianakis, 2016).

Soil texture and pH, unlike geography and soil cultivation, influenced the occurrence and abundance of representatives of the five different groups of bradyrhizobial root nodule symbionts with three groups responding to soil texture and two to soil pH (Fig. 4). The relative sand to clay content of the soils must have affected the bioavailability of P and other mineral nutrients in soil, water retention, as well as the number of habitats (soil pores and aggregates) for bacteria, protected against grazing protozoa (Ramirez and Alexander, 1980; van Veen et al., 1997). Clayey soils, such as those in the Kilifi area retain more water than sandy soils, which prevail around Mbeere. Clayey soils also support the survival of rhizobia by protecting them against high temperatures due to their composition of micro-aggregates (van Veen et al., 1997). Zengeni et al. (2006) demonstrated that the survival of rhizobia was poorer in soils low in clay. Soil pH has been found to influence bradyrhizobial diversity in cowpea and other legumes (Palmer and Young, 2000; Zhang et al., 2011; Cao et al., 2014; Wang et al., 2016), most likely, because the pH affects the bioavailability of mineral nutrients in soils. Clearly, further studies are needed with a more extensive site and strain sampling, preferably across edaphic, ecological and farming intensity gradients, to better characterise the factors influencing *Bradyrhizobium* diversity and abundance. Such information is vital for inoculation, since strains have to be chosen that establish and persist to become effective in stimulating plant growth.

### 4.4 Discrimination of bradyrhizobial root nodule isolates based on their protein profiles and 16S rRNA gene sequences

MALDI-TOF MS protein profiles and 16S rRNA gene sequences, both allowed grouping the root nodule symbionts of cowpea. These groupings were largely congruent, although the protein profile-based approach yielded some more resolution than the 16S rRNA gene-based phylogenetic discrimination. Both, the MALDI-TOF MS protein profile- and 16S rRNA gene-based approaches, similarly assigned 171 newly isolated bacterial strains of cowpea root nodules to the genus *Bradyrhizobium*. The protein profile-based approach revealed five clearly distinct clusters (Fig. 1). Correct assignment to the genus *Bradyrhizobium* by MALDI-TOF MS protein profiling was confirmed by sequencing the 16S rRNA gene of 25 representative strains of all five *Bradyrhizobium* clusters. These strains also originated from the two different agro-ecological regions and cultivated and uncultivated sites and references strains from a commercial inoculant produced in Kenya and an effective N_2_ fixing strain from Burkina Faso. Selection of these strains targeted to cover any possible spatial distributional heterogeneity, confirming the reliability of taxonomic assignment. This indicates that similarity grouping, based on mass spectral profiles of all cellular proteins in the Spectral ARchive And Microbial Identification System (SARAMIS™) with the Superspectra database can, indeed, confidently assign new isolates of *Bradyrhizobium* of cowpea to species represented in the database (Ziegler et al., 2015) (Fig. 1, Fig. 2, Table S2). Previous studies indicated that 16S rRNA gene sequencing lacks sufficient resolution to confidently delineate species within the genus *Bradyrhizobium* (Menna et al., 2009; Azevedo et al., 2015). 16S rRNA gene sequencing proofed, however, sufficient to resolve two super clades, corresponding to the species groups of *B. japonicum* and *B. elkanii*. Since, methodologically simple and showing some more resolution, the protein profiling approach was preferred for the aim of this study. Applying it to a collection of new bacterial isolates derived from nodules collected in the field and from trap cultures with field soil, this study confirmed that MALDI-TOF MS protein profiling can also be used on root nodule samples hosting yet unknown bacterial symbionts. It is operationally simpler, since just a one-step laboratory analytical procedure is needed (Ziegler et al., 2015), compared to PCR amplification of several genes (Menna et al., 2009; Delamuta et al., 2012; Wade et al., 2014; Grönemeyer et al., 2015a). MALDI-TOF MS protein profiling allows for high sample throughput at moderate costs without compromising on resolution. The method was already applied several times on rhizobia (Ferreira et al., 2011; Ziegler et al., 2012; Sanchez-Juanes et al., 2013; Ziegler et al., 2015; Fossou et al., 2016). It was for instance used to discriminate and detect *Bradyrhizobium* strains from nodules of *Lupinus* in Spain (Sanchez-Juanes et al., 2013) and most recently nodules of pigeonpea in Côte d’Ivoire (Fossou et al., 2016).

Previous studies on bradyrhizobia from Africa relied mostly on DNA-based discrimination methods, such as PCR-RFLP fingerprinting, 16S rRNA gene and ribosomal IGS single-marker or MLSA sequencing (Krasova-Wade et al., 2003; Wasike et al., 2009; Pule-Meulenberg et al., 2010; Mathu et al., 2012). Also other studies on legume-nodulating *Bradyrhizobia* from other parts of the world are, nowadays, relying on MLSA (Ormeño-Orrillo et al., 2006; Rivas et al., 2009; Delamuta et al., 2013; Delamuta et al., 2015). This study is, however, the first applying MALDI-TOF MS protein profiling in a field survey on cowpea-nodulating bradyrhizobia.

Yet, one limitation of MALDI-TOF MS-based protein profiling is that taxonomic assignment is only possible for taxa for which there is already information of reference strains in the database (Uhlik et al., 2011), as typical for fingerprinting/profiling approaches in microbial screening. Another limitation is that bacteria have to be isolated and cultured before MALDI-TOF based fingerprinting can be used. However, MALDI-TOF MS-based protein profiling can be used for simple strain discrimination as needed for most ecological investigations, once microbial isolates are available.

### 4.5 Next steps in rhizobial community analyses using protein profiles for discrimination

Besides extending the reference databases to improve taxonomic assignment, agreements should be reached about the level of resolution that is still reliable, given protein expression may vary, depending on the bacterial growth stage (symbiotic or saprobic; young or old), medium, host plant species etc. Furthermore, the reliability of the similarity clustering should be methodologically confirmed and eventually standard references defined to stabilize it as well as statistical methods found to support it. A further major developmental step will be to define sets of indicator proteins to deconvolute samples with several different strains. This would allow direct root nodule occupancy analyses and even analysis of pooled nodule samples, representative for entire plants, or entire fields. When this will be possible, protein profiling using MALDI-TOF MS could be used to link rhizobial community profiling to functional measures, such as plant growth, or nitrogen nutritional measurements on symbiotic N_2_ fixation. Furthermore, a deliberate focus on differentially translated rather than presence/absence of proteins depending on symbiotic efficiency would further support analyses on symbiotic functioning. A linking of metatranscriptomics and genomics data with the protein profiles could ultimately reveal distinctive metabolic functions.

## 5 Conclusions

This study showed that there are virtually no differences between the root nodule-colonising rhizobial communities of cowpea between contrasting agro-ecological regions and cultivated and uncultivated sites in Kenya including reference strains CBA from Biofix inoculant produced in Kenya and BK1 isolated from cowpea cultivated in soil from Burkina Faso. However, the richness of cowpea nodule symbionts was found to be high at each individual site. This may relate to the considerable promiscuity of cowpea for several different species of the genus *Bradyrhizobium* as well as, an apparent widespread distribution of the dominant symbionts. Yet more different and also some unique, but rare rhizobia, were found in the drier in- and upland agro-ecological region than the humid, coastland region and at uncultivated, compared to cultivated sites. We speculate that this may be explained by reduced protozoan predation in drier soils and higher plant species richness in the vegetation cover of uncultivated sites. Unlike geography, soil texture and pH influenced the occurrence and abundance of the resolved bradyrhizobial groups, pointing at a possibility to find suitable rhizobial inoculants for cowpea at sites with different soils to lower the dependence on mineral N fertilizer in efforts to maintain soil fertility and crop productivity.

MALDI-TOF MS protein profiling proofed applicable to the screening of new collections of unknown rhizobia from root nodules collected from plants that had been growing in the field, and such raised in trap cultures with soil samples from the field. In comparison with traditional 16S rRNA gene sequencing, MALDI-TOF MS protein profiling resolved more species groups and also allowed taxonomic assignment, provided the reference database contained information of sufficiently similar strains. Acknowledging its limitations, MALDI-TOF MS protein profiling may thus be suitable to trace known rhizobial inoculant strains in root nodules of field grown legumes.
