# Supplementary material for: Cowpea (Vigna unguiculata L. Walp) hosts several widespread bradyrhizobial root nodule symbionts across contrasting agro-ecological production areas in Kenya
Source: Agric Ecosyst Environ. 2018 Jul 1;261:161–71. doi: 10.1016/j.agee.2017.12.014 (PMC5946706; doi:10.1016/j.agee.2017.12.014)
Supplement: Supplementary file 1 [file mmc1.doc]

*Agriculture, Ecosystems & Environment*

**Cowpea (*Vigna unguiculata* L. Walp) hosts several widespread bradyrhizobial root nodule symbionts across contrasting agro-ecological production areas in Kenya**

Samuel Mathu Ndungu, Monika M. Messmer, Dominik Ziegler, Hannes A. Gamper, Éva Mészáros, Moses Thuita, Bernard Vanlauwe, Emmanuel Frossard and Cécile Thonar

**Supplementary material**

**Table S1.** Characteristics of the 40 study sites, including location, soil texture, cultivation status, and past and present cropping.

| **Site** | **Region** | **Coordinates** | | **Soil texture** | **Cultivation** | **Legume history previous 5 years** | **Duration of cowpea cultivation (years)** | **Intercrop of cowpea at sampling time** |
| --- | --- | --- | --- | --- | --- | --- | --- | --- |
| 1 | Mbeere | S 00^0^ 44.269′ | E 037^0^ 39.550′ | sandy loam | cultivated | cp, gg, cb | > 30 | millet, sorghum |
| 2 | Mbeere | S 00^0^ 44.443′ | E 037^0^ 39.421′ | sandy loam | cultivated | cp, pp, cb | > 30 | maize |
| 3 | Mbeere | S 00^0^ 45.172′ | E 037^0^ 39.406′ | sandy loam | cultivated | cp, gg, cb | > 20 | maize |
| 4 | Mbeere | S 00^0^ 45.824′ | E 037^0^ 39.726′ | sandy loam | cultivated | cp, gg, cb | > 40 | maize |
| 5 | Mbeere | S 00^0^ 45.577′ | E 037^0^ 39.894′ | sandy clay loam | cultivated | cp, gg, cb | > 40 | maize |
| 6 | Mbeere | S 00^0^ 46.176′ | E 037^0^ 39.654′ | sandy loam | cultivated | cp, gg, cb | 2 | maize |
| 7 | Mbeere | S 00^0^ 46.358′ | E 037^0^ 38.952′ | sandy loam | cultivated | cp, gg, cb | > 30 | maize |
| 8 | Mbeere | S 00^0^ 46.266′ | E 037^0^ 38.851′ | sandy clay loam | cultivated | cp, gg, cb | 9 | maize |
| 9 | Mbeere | S 00^0^ 46.920′ | E 037^0^ 39.141′ | sandy clay loam | cultivated | cp, gg, cb | > 30 | maize |
| 10 | Mbeere | S 00^0^ 47.182′ | E 037^0^ 39.080′ | sandy loam | cultivated | cp, gg, cb | > 40 | maize |
| 11 | Mbeere | S 00^0^ 46.952′ | E 037^0^ 40.752′ | sandy loam | cultivated | cp, gg, cb | > 40 | maize, cassava, sweet potato |
| 12 | Mbeere | S 00^0^ 46.735′ | E 037^0^ 40.626′ | sandy loam | cultivated | cp, gg, cb | > 30 | maize |
| 13 | Mbeere | S 00^0^ 46.137′ | E 037^0^ 40.193′ | sandy loam | cultivated | cp, gg, cb | 5 | maize |
| 14 | Mbeere | S 00^0^ 45.807′ | E 037^0^ 40.117′ | sandy clay loam | cultivated | cp, gg, pp, cb | > 20 | maize |
| 15 | Mbeere | S 00^0^ 45.441′ | E 037^0^ 40.218′ | sandy loam | cultivated | cp, gg, cb | 18 | maize, sorghum |
| 16 | Mbeere | S 00^0^ 45.064′ | E 037^0^ 39.292′ | sandy loam | uncultivated | None | n.a. | n.a. |
| 17 | Mbeere | S 00^0^ 45.617′ | E 037^0^ 39.954′ | clay loam | uncultivated | None | n.a. | n.a. |
| 18 | Mbeere | S 00^0^ 46.805′ | E 037^0^ 39.218′ | sandy clay loam | uncultivated | None | n.a. | n.a. |
| 19 | Mbeere | S 00^0^ 46.942′ | E 037^0^ 40.724′ | sandy loam | uncultivated | None | n.a. | n.a. |
| 20 | Mbeere | S 00^0^ 46.233′ | E 037^0^ 40.152′ | sandy loam | uncultivated | None | n.a. | n.a. |
| 21 | Kilifi | S 03^0^ 45.711′ | E 039^0^ 40.049′ | sandy loam | cultivated | cp | 18 | maize, cassava, cashew, coconut, mango |
| 22 | Kilifi | S 03^0^ 46.114′ | E 039^0^ 40.285′ | sand | cultivated | cp, gg, cb | 18 | maize, cassava |
| 23 | Kilifi | S 03^0^ 45.589′ | E 039^0^ 40.242′ | sandy loam | cultivated | cp | 4 | maize, cassava |
| 24 | Kilifi | S 03^0^ 45.752′ | E 039^0^ 40.290′ | loamy sand | cultivated | cp, gg, cb | 20 | maize, cassava |
| 25 | Kilifi | S 03^0^ 46.955′ | E 039^0^ 40.930′ | sandy clay loam | cultivated | cp and cb | 4 | maize, cassava |
| 26 | Kilifi | S 03^0^ 46.860′ | E 039^0^ 40.924′ | sandy loam | cultivated | cp and gg | 5 | maize, cassava |
| 27 | Kilifi | S 03^0^ 47.620′ | E 039^0^ 41.299′ | clay loam | cultivated | cp and gg | 10 | maize, cassava, pawpaw, orange |
| 28 | Kilifi | S 03^0^ 45.968′ | E 039^0^ 43.835′ | clay | cultivated | cp, gg, cb | > 20 | maize, cassava, okra |
| 29 | Kilifi | S 03^0^ 46.055′ | E 039^0^ 43.579′ | sandy clay loam | cultivated | cp and gg | 6 | maize, cassava |
| 30 | Kilifi | S 03^0^ 46.182′ | E 039^0^ 43.643′ | clay | cultivated | cp and gg | > 10 | maize, cassava |
| 31 | Kilifi | S 03^0^ 46.243′ | E 039^0^ 44.724′ | clay | cultivated | cp and gg | 4 | maize, cassava, okra |
| 32 | Kilifi | S 03^0^ 46.770′ | E 039^0^ 44.868′ | clay | cultivated | cp, gg, cb | 2 | maize, sorghum, banana, pawpaw |
| 33 | Kilifi | S 03^0^ 44.826′ | E 039^0^ 44.945′ | clay | cultivated | cp and gg | > 20 | maize, cassava |
| 34 | Kilifi | S 03^0^ 44.457′ | E 039^0^ 46.217′ | loam | cultivated | cp and gg | > 10 | maize, cashew, coconut, mango |
| 35 | Kilifi | S 03^0^ 44.275′ | E 039^0^ 46.398′ | loam | cultivated | cp and gg | 15 | maize, cassava |
| 36 | Kilifi | S 03^0^ 44.323′ | E 039^0^ 41.315′ | sandy loam | uncultivated | None | n.a. | n.a. |
| 37 | Kilifi | S 03^0^ 46.322′ | E 039^0^ 40.195′ | loamy sand | uncultivated | None | n.a. | n.a. |
| 38 | Kilifi | S 03^0^ 47.201′ | E 039^0^ 41.702′ | sandy clay | uncultivated | None | n.a. | n.a. |
| 39 | Kilifi | S 03^0^ 46.186′ | E 039^0^ 43.592′ | clay | uncultivated | None | n.a. | n.a. |
| 40 | Kilifi | S 03^0^ 46.490′ | E 039^0^ 44.843′ | clay | uncultivated | None | n.a. | n.a. |

n.a.: not applicable, cb: common bean (*Phaseolus vulgaris* L.), cp: cowpea (*Vigna unguiculata* L. Walp), gg: green gram (*Vigna radiata* (L.) Wilczek.), pp: pigeonpea (*Cajanus cajan* (L.) Millsp.), maize (*Zea mays* L.), sorghum (*Sorghum bicolor* (L.) Conrad Moench), pearl millet (*Pennisetum glaucum* (L.)R.Br), cassava (*Manihot esculenta* Crantz), sweet potato (*Ipomoea batatas* (L.) Lam), okra [*Abelmoschus esculentus* (L.) Moench], banana (*Musa sp.* L.), pawpaw (*Carica papaya* L.), coconut (*Cocos nucifera* L.), mango (*Mangifera indica* L.), cashew (*Anacardium occidentale* L.), orange (*Citrus sinensis* L.).

**Table S2. Origin and identity of rhizobial isolates of root nodules of cowpea, collected in the field and from trap cultures. Identities refer to similarity clusters as determined by MALDI-TOF MS analysis of the protein mass spectra of cells propagated on solid Modified Arabinose Gluconate medium (Sadows*ky et a*l., 1987; Van Berkum, 1990).**

| **Region** | **Strain** | **Site** | **MALDITOF ID** | **Cluster** | **Sub-cluster** |
| --- | --- | --- | --- | --- | --- |
| Mbeere | M1b | Cultivated | *Bradyrhizobium* cf. *japonicum* | 3 | 3c |
| Mbeere | M1c | Cultivated | *Bradyrhizobium* *elkanii* | 5 | 5c |
| Mbeere | M1d | Cultivated | *Bradyrhizobium* cf. *japonicum* | 3 | 3d |
| Mbeere | M1e | Cultivated | *Bradyrhizobium* cf. *diazoefficiens* | 1 | - |
| Mbeere | M1f | Cultivated | *Bradyrhizobium* sp. II | 3 | 3b |
| Mbeere | M1g | Cultivated | *Bradyrhizobium* cf. *japonicum* | 3 | 3e |
| Mbeere | M1h⃰ | Cultivated | *Bradyrhizobium* cf. *japonicum* | 3 | 3c |
| Mbeere | M1j | Cultivated | *Bradyrhizobium* cf. *japonicum* | 3 | 3e |
| Mbeere | M2b | Cultivated | *Bradyrhizobium* cf. *diazoefficiens* | 1 | - |
| Mbeere | M2e | Cultivated | *Bradyrhizobium* *elkanii* | 5 | 5c |
| Mbeere | M2h⃰ | Cultivated | *Bradyrhizobium* *elkanii* | 5 | 5c |
| Mbeere | M2i | Cultivated | *Bradyrhizobium* *elkanii* | 5 | 5c |
| Mbeere | M2j1 | Cultivated | *Bradyrhizobium* *elkanii* | 5 | 5c |
| Mbeere | M3c | Cultivated | *Bradyrhizobium* cf. *japonicum* | 3 | 3d |
| Mbeere | M3d | Cultivated | *Bradyrhizobium* cf. *japonicum* | 3 | 3b |
| Mbeere | M3e | Cultivated | *Bradyrhizobium* cf. *japonicum* | 3 | 3d |
| Mbeere | M3g | Cultivated | *Bradyrhizobium* cf. *japonicum* | 3 | 3e |
| Mbeere | M3h⃰ | Cultivated | *Bradyrhizobium* sp. II | 3 | 3b |
| Mbeere | M4a | Cultivated | *Bradyrhizobium* *elkanii* | 5 | 5c |
| Mbeere | M4d | Cultivated | *Bradyrhizobium* cf. *japonicum* | 3 | 3d |
| Mbeere | M4g | Cultivated | *Bradyrhizobium* cf. *japonicum* | 3 | 3d |
| Mbeere | M5a | Cultivated | *Bradyrhizobium* cf. *diazoefficiens* | unclustered | |
| Mbeere | M5b | Cultivated | *Bradyrhizobium* cf. *diazoefficiens* | unclustered | |
| Mbeere | M5d | Cultivated | *Bradyrhizobium* sp. II | 3 | 3b |
| Mbeere | M5e | Cultivated | *Bradyrhizobium* cf. *diazoefficiens* | 2 | - |
| Mbeere | M5f | Cultivated | *Bradyrhizobium* cf. *diazoefficiens* | 1 | - |
| Mbeere | M6b | Cultivated | *Bradyrhizobium* cf. *diazoefficiens* | 2 | - |
| Mbeere | M6c | Cultivated | *Bradyrhizobium* sp. I | 3 | 3a |
| Mbeere | M6d | Cultivated | *Bradyrhizobium* sp. III | 3 | 3a |
| Mbeere | M6e | Cultivated | *Bradyrhizobium* *elkanii* | 5 | 5c |
| Mbeere | M6f | Cultivated | *Bradyrhizobium* cf. *diazoefficiens* | unclustered | |
| Mbeere | M6g | Cultivated | *Bradyrhizobium* *elkanii* | 5 | 5c |
| Mbeere | M6h⃰ | Cultivated | *Bradyrhizobium* cf. *japonicum* | 3 | 3d |
| Mbeere | M6i | Cultivated | *Bradyrhizobium* cf. *diazoefficiens* | 2 | - |
| Mbeere | M6a | Cultivated | *Bradyrhizobium* cf. *diazoefficiens* | 1 | - |
| Mbeere | M6j | Cultivated | *Bradyrhizobium* sp. V | 3 | 3a |
| Mbeere | M7b | Cultivated | *Bradyrhizobium* cf. *diazoefficiens* | unclustered | |
| Mbeere | M7e | Cultivated | *Bradyrhizobium* cf. *japonicum* | 3 | 3e |
| Mbeere | M7f | Cultivated | *Bradyrhizobium* cf. *japonicum* | 3 | 3d |
| Mbeere | M7g | Cultivated | *Bradyrhizobium* cf. *japonicum* | 3 | 3d |
| Mbeere | M7h | Cultivated | *Bradyrhizobium* cf. *diazoefficiens* | unclustered | |
| Mbeere | M7i | Cultivated | *Bradyrhizobium* sp. VI | unclustered | |
| Mbeere | M7j | Cultivated | *Bradyrhizobium* cf. *japonicum* | 3 | 3d |
| Mbeere | M8b | Cultivated | *Bradyrhizobium* *elkanii* | 5 | 5c |
| Mbeere | M8e | Cultivated | *Bradyrhizobium* cf. *diazoefficiens* | 1 | - |
| Mbeere | M8g | Cultivated | *Bradyrhizobium* cf. *diazoefficiens* | 1 | - |
| Mbeere | M8i | Cultivated | *Bradyrhizobium* sp. VIII | unclustered | |
| Mbeere | M8a | Cultivated | *Bradyrhizobium* *elkanii* | 5 | 5a |
| Mbeere | M9c | Cultivated | *Bradyrhizobium* cf. *japonicum* | 3 | 3c |
| Mbeere | M9d | Cultivated | *Bradyrhizobium* cf. *japonicum* | 3 | 3e |
| Mbeere | M9h | Cultivated | *Bradyrhizobium* *elkanii* | 5 | 5c |
| Mbeere | M9i⃰ | Cultivated | *Bradyrhizobium* cf. *japonicum* | 3 | 3c |
| Mbeere | M9j | Cultivated | *Bradyrhizobium* cf. *diazoefficiens* | 1 | - |
| Mbeere | M10c | Cultivated | *Bradyrhizobium* sp. II | 3 | 3b |
| Mbeere | M10f | Cultivated | *Bradyrhizobium* cf. *diazoefficiens* | unclustered | |
| Mbeere | M10g | Cultivated | *Bradyrhizobium* cf. *diazoefficiens* | unclustered | |
| Mbeere | M10j | Cultivated | *Bradyrhizobium* cf. *Japonicum* | 3 | 3b |
| Mbeere | M11c⃰ | Cultivated | *Bradyrhizobium* cf. *diazoefficiens* | unclustered | |
| Mbeere | M11h | Cultivated | *Bradyrhizobium* sp. VII | unclustered | |
| Mbeere | M12d | Cultivated | *Bradyrhizobium* cf. *diazoefficiens* | unclustered | |
| Mbeere | M12e | Cultivated | *Bradyrhizobium* cf. *diazoefficiens* | 1 | - |
| Mbeere | M12f⃰ | Cultivated | *Bradyrhizobium* *elkanii* | 5 | 5c |
| Mbeere | M13e | Cultivated | *Bradyrhizobium* cf. *japonicum* | 3 | 3c |
| Mbeere | M13j1⃰ | Cultivated | *Bradyrhizobium* sp. IV | unclustered | |
| Mbeere | M14b | Cultivated | *Bradyrhizobium* cf. *diazoefficiens* | 1 | - |
| Mbeere | M14d | Cultivated | *Bradyrhizobium* cf. *diazoefficiens* | 1 | - |
| Mbeere | M17b | Uncultivated | *Bradyrhizobium* *elkanii* | 5 | 5c |
| Mbeere | M17c | Uncultivated | *Bradyrhizobium* *elkanii* | 5 | 5c |
| Mbeere | M17e | Uncultivated | *Bradyrhizobium* *elkanii* | 5 | - |
| Mbeere | M18a | Uncultivated | *Bradyrhizobium* cf. *diazoefficiens* | 1 | - |
| Mbeere | M18f⃰ | Uncultivated | *Bradyrhizobium* *elkanii* | 5 | 5c |
| Mbeere | M19c⃰ | Uncultivated | *Bradyrhizobium* sp. V | 3 | 3a |
| Mbeere | M19e | Uncultivated | *Bradyrhizobium* *elkanii* | 5 | 5c |
| Mbeere | M20a⃰ | Uncultivated | *Bradyrhizobium* cf. *diazoefficiens* | 1 | - |
| Mbeere | M20b | Uncultivated | *Bradyrhizobium* cf. *diazoefficiens* | 2 | - |
| Mbeere | M20c | Uncultivated | *Bradyrhizobium* *elkanii* | 5 | 5a |
| Mbeere | M20d | Uncultivated | *Bradyrhizobium* *elkanii* | 5 | 5c |
| Mbeere | M20e | Uncultivated | *Bradyrhizobium* *elkanii* | 5 | 5c |
| Kilifi | K1g | Cultivated | *Bradyrhizobium* *elkanii* | 5 | 5e |
| Kilifi | K1h⃰ | Cultivated | *Bradyrhizobium* *elkanii* | 5 | 5a |
| Kilifi | K1i | Cultivated | *Bradyrhizobium* *elkanii* | 5 | 5e |
| Kilifi | K2g | Cultivated | *Bradyrhizobium* *elkanii* | 5 | 5b |
| Kilifi | K3a | Cultivated | *Bradyrhizobium* *elkanii* | 5 | 5b |
| Kilifi | K3b | Cultivated | *Bradyrhizobium* cf. *diazoefficiens* | unclustered | |
| Kilifi | K3c⃰ | Cultivated | *Bradyrhizobium* *elkanii* | 5 | 5e |
| Kilifi | K3f | Cultivated | *Bradyrhizobium* cf. *diazoefficiens* | 1 | - |
| Kilifi | K3g | Cultivated | *Bradyrhizobium* *elkanii* | 5 | 5e |
| Kilifi | K3j | Cultivated | *Bradyrhizobium* *elkanii* | 5 | 5b |
| Kilifi | K4b | Cultivated | *Bradyrhizobium* *elkanii* | 5 | 5b |
| Kilifi | K6b1 | Cultivated | *Bradyrhizobium* *elkanii* | 5 | 5b |
| Kilifi | K6d | Cultivated | *Bradyrhizobium* cf. *diazoefficiens* | 2 | - |
| Kilifi | K6e⃰ | Cultivated | *Bradyrhizobium* cf. *diazoefficiens* | 2 | - |
| Kilifi | K6j | Cultivated | *Bradyrhizobium* cf. *diazoefficiens* | 2 | - |
| Kilifi | K7a⃰ | Cultivated | *Bradyrhizobium* sp. I | 4 | - |
| Kilifi | K7e | Cultivated | *Bradyrhizobium* *elkanii* | 5 | 5d |
| Kilifi | K7b | Cultivated | *Bradyrhizobium* cf. *diazoefficiens* | 1 | - |
| Kilifi | K7c | Cultivated | *Bradyrhizobium* *elkanii* | 5 | 5d |
| Kilifi | K7d | Cultivated | *Bradyrhizobium* cf. *diazoefficiens* | 1 | - |
| Kilifi | K8a | Cultivated | *Bradyrhizobium* cf. *diazoefficiens* | 1 | - |
| Kilifi | K8b | Cultivated | *Bradyrhizobium* *elkanii* | 5 | 5d |
| Kilifi | K8e | Cultivated | *Bradyrhizobium* *elkanii* | 5 | 5d |
| Kilifi | K9d | Cultivated | *Bradyrhizobium* cf. *japonicum* | 3 | 3e |
| Kilifi | K9f⃰ | Cultivated | *Bradyrhizobium* cf. *diazoefficiens* | 1 | - |
| Kilifi | K9g | Cultivated | *Bradyrhizobium* cf. *japonicum* | 3 | 3d |
| Kilifi | K9h | Cultivated | *Bradyrhizobium* *elkanii* | 5 | 5b |
| Kilifi | K10a | Cultivated | *Bradyrhizobium* cf. *diazoefficiens* | 1 | - |
| Kilifi | K10b | Cultivated | *Bradyrhizobium* cf. *japonicum* | 3 | 3e |
| Kilifi | K10c | Cultivated | *Bradyrhizobium* cf. *japonicum* | 3 | 3e |
| Kilifi | K10e⃰ | Cultivated | *Bradyrhizobium* cf. *japonicum* | 3 | 3d |
| Kilifi | K10f | Cultivated | *Bradyrhizobium* cf. *japonicum* | 3 | 3d |
| Kilifi | K10h | Cultivated | *Bradyrhizobium* cf. *diazoefficiens* | 3 | 3c |
| Kilifi | K10i | Cultivated | *Bradyrhizobium* cf. *japonicum* | 3 | 3d |
| Kilifi | K10j | Cultivated | *Bradyrhizobium* cf. *japonicum* | 3 | 3e |
| Kilifi | K11a | Cultivated | *Bradyrhizobium* sp. I | 4 | - |
| Kilifi | K11d | Cultivated | *Bradyrhizobium* cf. *diazoefficiens* | 1 | - |
| Kilifi | K11e | Cultivated | *Bradyrhizobium* *elkanii* | 5 | 5b |
| Kilifi | K11f | Cultivated | *Bradyrhizobium* *elkanii* | 5 | 5b |
| Kilifi | K11g | Cultivated | *Bradyrhizobium* sp. I | 4 | - |
| Kilifi | K11h | Cultivated | *Bradyrhizobium* *elkanii* | 5 | 5b |
| Kilifi | K11j | Cultivated | *Bradyrhizobium* sp. I | 4 | - |
| Kilifi | K12g | Cultivated | *Bradyrhizobium* *elkanii* | 5 | 5e |
| Kilifi | K12h | Cultivated | *Bradyrhizobium* cf. *diazoefficiens* | 2 | - |
| Kilifi | K12i | Cultivated | *Bradyrhizobium* sp. I | 4 | - |
| Kilifi | K12j | Cultivated | *Bradyrhizobium* sp. I | 4 | - |
| Kilifi | K13a | Cultivated | *Bradyrhizobium* cf. *japonicum* | 3 | 3d |
| Kilifi | K13b | Cultivated | *Bradyrhizobium* sp. I | 4 | - |
| Kilifi | K13c | Cultivated | *Bradyrhizobium* *elkanii* | 5 | 5b |
| Kilifi | K13d | Cultivated | *Bradyrhizobium* sp. I | 4 | - |
| Kilifi | K13e | Cultivated | *Bradyrhizobium* cf. *japonicum* | 3 | 3d |
| Kilifi | K13f | Cultivated | *Bradyrhizobium* cf. *diazoefficiens* | unclustered | |
| Kilifi | K13g | Cultivated | *Bradyrhizobium* sp. I | 4 | - |
| Kilifi | K13h | Cultivated | *Bradyrhizobium* sp. I | 4 | - |
| Kilifi | K13j | Cultivated | *Bradyrhizobium* sp. I | 4 | - |
| Kilifi | K14a | Cultivated | *Bradyrhizobium* cf. *diazoefficiens* | 2 | - |
| Kilifi | K14b | Cultivated | *Bradyrhizobium* *elkanii* | 5 | 5b |
| Kilifi | K14d | Cultivated | *Bradyrhizobium* cf. *diazoefficiens* | 2 | - |
| Kilifi | K14e | Cultivated | *Bradyrhizobium* *elkanii* | 5 | 5d |
| Kilifi | K14f | Cultivated | *Bradyrhizobium* cf. *diazoefficiens* | 1 | - |
| Kilifi | K14g | Cultivated | *Bradyrhizobium* *elkanii* | 5 | 5b |
| Kilifi | K14h⃰ | Cultivated | *Bradyrhizobium* cf. *diazoefficiens* | 1 | - |
| Kilifi | K15b | Cultivated | *Bradyrhizobium* cf. *diazoefficiens* | 1 | - |
| Kilifi | K15c⃰ | Cultivated | *Bradyrhizobium* *elkanii* | 5 | 5e |
| Kilifi | K15d | Cultivated | *Bradyrhizobium* *elkanii* | 5 | 5b |
| Kilifi | K15e | Cultivated | *Bradyrhizobium* *elkanii* | 5 | 5a |
| Kilifi | K15f | Cultivated | *Bradyrhizobium* *elkanii* | 5 | 5b |
| Kilifi | K15g | Cultivated | *Bradyrhizobium* *elkanii* | 5 | 5b |
| Kilifi | K16a | Uncultivated | *Bradyrhizobium* *elkanii* | 5 | 5a |
| Kilifi | K16b | Uncultivated | *Bradyrhizobium* *elkanii* | 5 | 5b |
| Kilifi | K16c⃰ | Uncultivated | *Bradyrhizobium* cf. *diazoefficiens* | 2 | - |
| Kilifi | K16e | Uncultivated | *Bradyrhizobium* cf. *diazoefficiens* | 2 | - |
| Kilifi | K17a | Uncultivated | *Bradyrhizobium* *elkanii* | 5 | 5b |
| Kilifi | K17b | Uncultivated | *Bradyrhizobium* *elkanii* | 5 | 5b |
| Kilifi | K17c | Uncultivated | *Bradyrhizobium* *elkanii* | 5 | 5b |
| Kilifi | K17d | Uncultivated | *Bradyrhizobium* *elkanii* | 5 | 5d |
| Kilifi | K17e | Uncultivated | *Bradyrhizobium* cf. *diazoefficiens* | 1 | - |
| Kilifi | K17f⃰ | Uncultivated | *Bradyrhizobium* *elkanii* | 5 | 5b |
| Kilifi | K17g | Uncultivated | *Bradyrhizobium* *elkanii* | 5 | 5b |
| Kilifi | K18a | Uncultivated | *Bradyrhizobium* cf. *japonicum* | 3 | 3d |
| Kilifi | K18b | Uncultivated | *Bradyrhizobium* cf. *japonicum* | 3 | 3b |
| Kilifi | K18c | Uncultivated | *Bradyrhizobium* cf. *diazoefficiens* | 2 | - |
| Kilifi | K18e | Uncultivated | *Bradyrhizobium* cf. *japonicum* | 3 | 3b |
| Kilifi | K18f | Uncultivated | *Bradyrhizobium* cf. *diazoefficiens* | 2 |  |
| Kilifi | K19a⃰ | Uncultivated | *Bradyrhizobium* cf. *japonicum* | 3 | 3f |
| Kilifi | K19b | Uncultivated | *Bradyrhizobium* cf. *japonicum* | 3 | 3f |
| Kilifi | K19c | Uncultivated | *Bradyrhizobium* cf. *japonicum* | 3 | 3d |
| Kilifi | K20a | Uncultivated | *Bradyrhizobium* cf. *japonicum* | 3 | 3d |
| Kilifi | K20b | Uncultivated | *Bradyrhizobium* cf. *japonicum* | 3 | 3d |
| Kilifi | K20d | Uncultivated | *Bradyrhizobium* cf. *japonicum* | 3 | 3c |
| Kilifi | K20f⃰ | Uncultivated | *Bradyrhizobium* cf. *diazoefficiens* | 3 | 3c |
| Kilifi | K20h | Uncultivated | *Bradyrhizobium* cf. *diazoefficiens* | 3 | 3c |
| Kilifi | K20i | Uncultivated | *Bradyrhizobium* sp. I | 4 | - |
| Biofix | CBA⃰ | Biofix CB1015 | *Bradyrhizobium* cf*. diazoefficiens* | 3 | - |
| Burkina | BK1⃰ | Burkina nodules | *Bradyrhizobium sp.* III | 3 | 3a |
| Mbeere | M1a | Cultivated | *Enterobacter cloacae* | - | - |
| Mbeere | M1i2 | Cultivated | *Rhizobium radiobacter* | - | - |
| Mbeere | M2a | Cultivated | *Rhizobium sp.* | - | - |
| Mbeere | M2c | Cultivated | *Unknown* | - | - |
| Mbeere | M2d | Cultivated | *Unknown* | - | - |
| Mbeere | M2g | Cultivated | *Rhizobium sp.* | - | - |
| Mbeere | M4e | Cultivated | *Unknown* | - | - |
| Mbeere | M5j2 | Cultivated | *Unknown* | - | - |
| Mbeere | M9b | Cultivated | *Rhizobium radiobacter* | - | - |
| Mbeere | M10i | Cultivated | *Rhizobium radiobacter* | - | - |
| Mbeere | M12i | Cultivated | *Rhizobium sp.* | - | - |
| Mbeere | M13j2 | Cultivated | *Unknown* | - | - |
| Mbeere | M15a | Cultivated | *Rhizobium radiobacter* | - | - |
| Mbeere | M15f | Cultivated | *Unknown* | - | - |
| Kilifi | K1b | Cultivated | *Unknown* | - | - |
| Kilifi | K3h | Cultivated | *Unknown* | - | - |
| Kilifi | K5e | Cultivated | *Enterobacter cloacae* | - | - |
| Kilifi | K6h | Cultivated | *Unknown* | - | - |
| Kilifi | K8c | Cultivated | *Rhizobium radiobacter* | - | - |
| Kilifi | K9a | Cultivated | *Rhizobium sp.* | - | - |
| Kilifi | K9b | Cultivated | *Rhizobium sp.* | - | - |
| Kilifi | K11i | Cultivated | *Rhizobium radiobacter* | - | - |
| Kilifi | K19d | Uncultivated | *Enterobacter cloacae* | - | - |
| Kilifi | K20g | Uncultivated | *Staphylococcus warneri* | - | - |

Strains characterized using the presence/absence of characteristic protein masses in the size range of 3,000-12,000 Da. Binary matrices were generated for each bacterial strain and taxonomic assignment made by comparison to the Spectral ARchive And Microbial Identification System (SARAMIS^TM^), using the Superspectra tool, which relies on multivariate neighbor joining of Dice distances to find matches between the mass spectral protein profiles of unknown and reference strains (Ziegl*er et a*l., 2015). Asterisks (*) indicate those strains selected for further 16S rRNA gene sequencing (Fig. 2) for DNA-based phylotaxonomic assignment. Strains that did not cluster in any of the five clusters were affiliated to *Bradyrhizobium* cf. *diazoefficiens*, *Bradyrhizobium* sp. V, VI, VII and VIII of the identification system of Ziegl*er et a*l. (2015)
